# Public perceptions of quarantine: community-based telephone survey following an infectious disease outbreak

**DOI:** 10.1186/1471-2458-9-470

**Published:** 2009-12-16

**Authors:** C Shawn Tracy, Elizabeth Rea, Ross EG Upshur

**Affiliations:** 1 Primary Care Research Unit, Sunnybrook Health Sciences Centre, 2075 Bayview Avenue, Room E3-49, Toronto, Ontario, M4N 3M5, Canada; 2 Joint Centre for Bioethics, University of Toronto, 88 College Street, Toronto, Ontario, M5G 1L4, Canada; 3 Dalla Lana School of Public Health, University of Toronto, Health Sciences Building, 155 College Street, 6th Floor, Toronto, Ontario, M5T 3M7, Canada; 4 Toronto Public Health, 277 Victoria Street, 5th Floor, Toronto, Ontario, M5B 1W2, Canada; 5 Department of Family and Community Medicine, University of Toronto, 263 McCaul Street, 3rd Floor, Toronto, Ontario, M5T 1W7, Canada

## Abstract

**Background:**

The use of restrictive measures such as quarantine draws into sharp relief the dynamic interplay between the individual rights of the citizen on the one hand and the collective rights of the community on the other. Concerns regarding infectious disease outbreaks (SARS, pandemic influenza) have intensified the need to understand public perceptions of quarantine and other social distancing measures.

**Methods:**

We conducted a telephone survey of the general population in the Greater Toronto Area in Ontario, Canada. Computer-assisted telephone interviewing (CATI) technology was used. A final sample of 500 individuals was achieved through standard random-digit dialing.

**Results:**

Our data indicate strong public support for the use of quarantine when required and for serious legal sanctions against those who fail to comply. This support is contingent both on the implementation of legal safeguards to protect against inappropriate use and on the provision of psychosocial supports for those affected.

**Conclusion:**

To engender strong public support for quarantine and other restrictive measures, government officials and public health policy-makers would do well to implement a comprehensive system of supports and safeguards, to educate and inform frontline public health workers, and to engage the public at large in an open dialogue on the ethical use of restrictive measures during infectious disease outbreaks.

## Background

Long considered an anachronism from a bygone era, quarantine has re-emerged in the 21^st ^century as an important (albeit controversial) tool in the battle against infectious disease. Prior to the 2003 outbreak of severe acute respiratory syndrome (SARS), it had been more than 50 years since mass quarantine measures had been invoked in North America [1]. The SARS containment measures imposed in Canada and Asia, and on a lesser scale in the U.S., provoked a heated debate within the public health community regarding the ethics and legality of quarantine [2-7].

Likewise, the SARS experience has sparked a renewed research interest in the ethics and effectiveness of quarantine. The findings of two recent retrospective studies of the 1918 Spanish flu pandemic strongly suggest that it was non-pharmaceutical inventions such as quarantine and other social distancing measures that were most effective in slowing the rate of spread and minimizing the rate of death [8,9]. And data from SARS-affected regions have pointed to the enduring value and effectiveness of quarantine and other restrictive measures [10,11]. In contrast, there are those who argue that the use of quarantine during SARS was both ineffective and inefficient [6,7]. The advent of advanced statistical modelling has added a new dimension to this long-running debate [12,13].

Toronto experienced the largest outbreak of SARS in North America, with investigation of 2,132 potential cases and identification of 23,103 contacts of SARS patients who required quarantine [11]. Post-SARS investigations have detected myriad adverse effects among those quarantined: significant feelings of uncertainty, anxiety, and isolation [14]; experience of stigma, fear, and frustration [15]; symptoms of depression and post-traumatic stress disorder [16]; and loss of anonymity [17].

Despite the long and controversial history of quarantine, little is known about lay perceptions of and attitudes toward its modern-day use. In view of the evidence of potential adverse effects on individual well-being and psychosocial health, and owing to the critical necessity of high compliance in the event of a major infectious disease outbreak, it is increasingly important to understand how quarantine is perceived by the general public. Therefore, the objective of the present study was to determine prevailing public attitudes toward the use of quarantine as a means of infectious disease control.

## Methods

### Participants and setting

The study was conducted in two regions of the Greater Toronto Area (GTA), specifically the City of Toronto proper and the Regional Municipality of York located directly to the north of Toronto. The GTA is among the largest metropolitan areas in North America with a population exceeding 5.5 million [18]. As the urban centre of the GTA, the City of Toronto is a densely-populated, cosmopolitan city (population estimate: 2,500,000; population density: 3,972/km^2^; visible minority population: 46%). In contrast, York is a much less-densely populated suburban region comprised of several small cities and towns (population estimate: 900,000; population density: 506.7/km^2^; visible minority population: 30%).

The study sample was stratified to include an equal number of participants from Toronto and York. There was no age or gender stratification. All participants provided verbal consent over the telephone prior to the survey interview. Research ethics approval was obtained from the University of Toronto, Toronto Public Health, and York Region Public Health Unit.

### Survey instrument

The survey instrument was developed by Toronto Public Health for use in a telephone survey of the general public following the SARS outbreak. The data reported in this paper are derived from a subset of 15 survey items specifically designed to measure public attitudes towards the use of quarantine during infectious disease outbreaks. These items addressed issues ranging from the legality of restrictive measures, the perceived effectiveness of quarantine, and the supports that should be supplied to those affected by quarantine orders. Respondents were asked to indicate their level of agreement/disagreement with each item; the response format was a 5-point Likert-type design (1 = "Strongly Disagree"; 2 = "Somewhat Disagree"; 3 = "Neutral"; 4 = "Somewhat Agree"; and 5 = "Strongly Agree").

After the response format was explained and before the first survey item was asked, all participants were provided standardized definitions of 'quarantine' [*"Quarantine means that you must stay in a separate area away from others because you were around someone with a serious illness and so you might have it, too."*] and 'infectious disease' [*"Infectious disease means a sickness that you can catch from another person, like the flu or tuberculosis*.]. At the conclusion of the survey, respondents were asked to supply general demographic information.

### Data collection and analysis

Data collection occurred between April 25, 2005 and May 16, 2005. The survey was administered using computer-assisted telephone interviewing (CATI) technology. The 15 interviewers received training in advance and worked with the assistance of two project supervisors.

Potential participants were screened for eligibility at the beginning of each call. Inclusion criteria included the following: minimum age of 18 years, primary residence located within the study area during the SARS outbreak, English comprehension skills, and ability to provide informed consent. Those who did not meet the minimum age criteria were asked if another member of the household aged 18 or above was available to participate in the survey. A final sample of 500 individuals was achieved through standard random-digit dialing.

The survey response rate varied slightly by study region. Excluding calls to ineligible participants (i.e., did not meet inclusion criteria) and disqualified numbers (e.g., not in service, wrong number, fax/computer/business line), the final response rate was 27% for the City of Toronto and 31% for York Region.

A factor analysis using Varimax rotation with Kaiser Normalization was performed on the data yielding four factors. Composite index scores were then computed for each factor by summing the responses on items loading on the respective factors. Thus, if a factor comprised five items then individual composite scores for that index could range from 5 to 25.

Bi-variate and multivariate analyses were performed to investigate the inter-relationships among variables. All analyses were performed using SPSS 11.0 for Windows. No statistical weighting of the data was performed.

## Results

### Descriptive analysis

A total of 500 participants were administered the subset of survey items on quarantine. Table 1 presents a summary of the demographic characteristics of this sample. The majority were middle-aged (56%) and female (64%). Within this sample, 4% of participants were personally impacted by quarantine during the SARS crisis (i.e., either they or someone else in their home was ordered into quarantine).

**Table 1:** Demographic profile of survey respondents

	Gender		Total
	Female	Male	
**Personal Characteristics**			
*Age group*			
18-35 yrs	94	55	149
36-65 yrs	176	104	280
>65 yrs	51	19	70
Total	321	178	499
			
*Location*			
Toronto	153	97	250
York	169	81	250
Total	322	178	500
			
**Quarantine Status**			
*Was anyone in your home quarantined during SARS?*			
No	307	172	479
Yes, myself but nobody else in my home	8	1	9
Yes, myself and someone else in my home	5	3	8
Yes, not myself but someone else in my home	2	1	3
Total	322	177	499

Table 2 presents the distribution of responses for each of the 15 Likert-type survey items (from "Strongly Agree" through to "Strongly Disagree"). In the table, the wording of the individual items is precisely as appeared on the survey instrument; however, for the purposes of presentation, the items are clustered according to the findings of the factor analysis (as described below). As there were no significant differences between respondents from Toronto versus York, the overall results are shown. The vast majority of respondents indicated agreement (either "Strongly Agree" or "Somewhat Agree") that sufficient justification exists for the use of quarantine during infectious disease outbreaks. Similarly, most respondents agreed that public health authorities and government officials should endeavour to lessen the burdens endured by those ordered into quarantine. Likewise, there was majority support for the use of various legal sanctions, penalties, and/or coercive measures in order to maximize compliance with quarantine orders. And, finally, the vast majority of respondents were in favour of safeguards against unwarranted and/or inappropriate use of quarantine. While these high percentages suggest a certain degree of convergence of opinion, it is important to note that the proportion of respondents indicating "Strongly Agree" versus "Somewhat Agree" varies significantly across the 15 items, as indicated in Table 2.

**Table 2:** Public attitudes toward quarantine (Qx) by factor

	Strongly Agree	Somewhat Agree	Neutral	Somewhat Disagree	Strongly Disagree
**Justification**					
Public Health should have the power to order people into Qx during outbreaks	77%	18%	3%	1%	0%
Qx is a good way to stop the spread of infectious disease outbreaks	76%	18%	3%	3%	0%
If someone is given a Qx order by Public Health, they should follow it no matter what else is going on in their life at work or home	70%	22%	5%	2%	1%
If I go into Qx, my family/friends/community will be protected from becoming sick	66%	22%	4%	5%	3%
**Sanctions**					
People who break Qx orders on purpose should face legal penalties like a fine or jail	53%	25%	14%	4%	3%
Public Health should be able to lock people up if they fail to obey Qx orders	28%	30%	19%	11%	12%
Public Health should use electronic bracelets and in-home surveillance cameras for people who disobey Qx orders	27%	23%	20%	12%	18%
**Burdens**					
Public Health needs to explain to everyone why they should be allowed to use Qx	84%	13%	2%	0%	1%
Government should pay for nurses and counselors to help people who are in Qx	77%	16%	4%	2%	1%
Public Health should ensure that people have food and shelter while in Qx, and pay for it with public money if need be	68%	19%	7%	4%	3%
Government should pay for counselors and support groups so that people coming out of Qx have someone to talk to about it	43%	29%	14%	9%	6%
People in Qx should get money from the government to pay for missed time at work	43%	26%	17%	9%	6%
**Safeguards**					
Public Health should ensure that there is no discrimination in the use of Qx	91%	8%	1%	0%	0%
It is reasonable for some rights to be taken away during an infectious disease outbreak	52%	30%	8%	4%	6%
People who disagree with their Qx order should be able to request a review to have it ended early	43%	35%	10%	3%	9%

Finally, survey participants were asked to indicate, by way of forced choice, their response to this statement: "Breaking or not obeying a quarantine order is most like which of the following [choose 1 only]: (a) parking in a no-parking zone; (b) driving way above the speed limit on a busy street; or (c) physical assault." Fully 59% responded that breaking quarantine is most like 'physical assault,' whereas 27% selected 'driving above the speed limit' and 8% chose 'parking in a no-parking zone' (6% did not answer).

### Factor analysis

Principal components factor analysis of the survey data yielded an underlying factor structure of four independent factors. Based on a subjective analysis of the content of items loading on each individual factor, the four factors were labelled as follows: 'Justification,' 'Sanctions,' 'Burdens,' and 'Safeguards' (as shown in Table 2).

### Bivariate/multivariate analysis

Four sub-scales were computed by summing scores for the items within each of the factors identified in the factor analysis. In addition, a total composite index was computed by summing scores across the four sub-scales. Scores on the four sub-scales and composite scale were submitted to age, gender, and regional analysis.

Analysis of variance testing revealed a number of statistically significant age and gender differences. On the 'Justification' sub-scale, female respondents scored significantly higher than males [F = 11.456 (df = 1), p < .001], thereby indicating greater agreement that the use of quarantine is justified in the context of an infectious disease outbreak. With respect to age, older respondents (>65 yrs) indicated greater agreement that use of quarantine is justified than did the young (18-35 yrs) [F = 4.514 (df = 2), p < .01]. Also, older respondents agreed more strongly that the use of sanctions for quarantine absconders is appropriate when compared both with the young and with the middle-aged (36-65 yrs) [F = 4.577 (df = 2), p < .01]. There were no significant differences by region.

## Discussion

### Major findings

The quarantine of exposed persons (along with the isolation of infected persons) has been properly described as the most complex and most ethically and legally controversial intervention within the jurisdiction of public health [19]. Complexity and controversy notwithstanding, the present data indicate a very high rate of public acceptance of quarantine as a means to control the spread of infectious disease. Indeed, the vast majority of respondents indicated strong support for the use of quarantine in an infectious disease outbreak, for legal penalties against absconders, for social supports for those affected, and for public safeguards against potential inappropriate use.

Data on public attitudes toward quarantine in the wake of SARS are scarce. Public opinion polls have indicated high levels of acceptance of quarantine among samples of Toronto-area residents (97%) and US citizens (93%) [20]. These findings are supported by an observed non-compliance rate of only 0.1% among Torontonians requiring quarantine during SARS [11]. A qualitative study of factors influencing compliance with quarantine in Toronto identified 'protection of the health of the community' as a prominent motivating factor. The authors of the study concluded that "while the overall compliance rate among residents of the GTA appears to have been high, the influence of 'civic duty' and social responsibility may not be as significant in other countries and cultures" [21].

Comparative data from international studies do lend support to the theory that cultural values and societal norms impact upon quarantine compliance rates. Researchers at the Harvard School of Public Health and the U.S. Centers for Disease Control and Prevention surveyed residents of Hong Kong, Taiwan, Singapore, and the U.S. and found significant regional variability [22]. The proportion of survey respondents favouring the quarantine of persons suspected of having been exposed to a serious contagious disease was as follows: 76% in the U.S., 81% in Hong Kong, 89% in Singapore, and 95% in Taiwan. By way of comparison, in the present study, 94% of respondents agreed that quarantine is a good way to stop the spread of infectious disease outbreaks. Interestingly, in our study, significantly fewer (58%) agreed that public health officials should be able to detain those who fail to obey quarantine orders. Likewise, in the Harvard study, the proportions favouring the use of quarantine decrease significantly if people could be arrested for refusing (to a low of only 42% in the U.S. to a high of 70% in Taiwan). The authors partially attributed the observed differences to prior experience with infectious disease outbreaks in which quarantine and other restrictive measures were implemented [21].

In view of this inter-region variability, it is not surprising that the global community of public health experts is itself conflicted about the use of quarantine and other restrictive measures that impinge upon the intrinsic rights of individuals. Those who favour the consideration of quarantine during infectious disease outbreaks maintain that it is prudent public health policy [23], whereas those in opposition argue that quarantine is inherently paternalistic and an unnecessary breach of basic human rights [24]. Despite the difference of opinion, however, there does appear to be general agreement on this: "ultimately, public health must rely not on force but on persuasion, and not on blind trust but on trust based on transparency, accountability, democracy, and human rights" [24].

**Figure 1:**
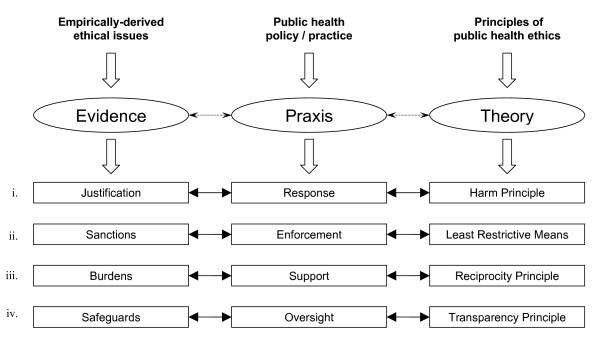
**An emerging conceptual framework for the ethical use of restrictive measures**.

With a view to fostering further deliberation and constructive debate, we are proposing a conceptual framework for the ethical use of restrictive measures in public health emergencies (see Figure 1). Building upon previous theoretical work on the justification for public health intervention [25], our model is designed to reflect the dynamic interplay among theory, empirical evidence, and policy/practice that is inherent to public health. To that end, we have incorporated the empirical data from the public opinion survey described in this paper. The model explicitly contemplates the four primary functions of public health as regards the use of restrictive measures in infectous disease outbreaks, namely, response, enforcement, support, and oversight. For instance, with respect to the enforcement of quarantine orders, the model illustrates how the specific function of enforcement aligns with the ethical principle of the 'least restrictive means' and is likewise concordant with empirical evidence indicating strong public support for the use of sanctions to promote compliance with quarantine orders (survey data reported here). This conceptual framework for the ethical use of restrictive measures in public health emergencies should be considered provisional and, as such, is open to further testing and refinement.

### Implications

Much has been learned from the unexpected arrival of SARS in the spring of 2003 [26,27]. Likewise, we continue to learn from historical analyses of the 1918 influenza pandemic, with one recent study providing strong support for the hypothesis that early implementation of public health measures such as quarantine can significantly reduce influenza transmission [9]. Given the current threat posed by pandemic influenza, it is incumbent upon the public health community-including ethicists and legal experts-to delineate both the limits to individual liberty and the obligations of public health authorities in the context of an infectious disease outbreak. It is noteworthy that the concept of 'voluntary quarantine' features prominently in many of the current plans for pandemic influenza. As contrasted with the classic quarantine order, which is typically enforceable by law, voluntary household quarantine refers to compliance based on the individual's own free will without legal compulsion.

Owing to the global threat of pandemic influenza, considerable planning and preparation for infectious disease outbreaks has been undertaken [28]. There remains a pressing need, however, to engage the citizenry more fully in the process of preparedness planning in order to ensure that the plans reflect the common will and that the policies serve the common good [29]. In this regard, the continuing growth in interest and activity in the subfield of public health ethics is certainly welcome and holds greats promise.

While we believe the data reported here contribute to the goal of better planning and better preparedness, the present study is limited by its sample of respondents who were drawn only from the Greater Toronto Area. Our goal was to assess the attitudes and perceptions of those living in an area significantly impacted by the SARS outbreak, but further research is now required to determine the generalizability of the present findings to other geographic regions and other populations. Also, our survey was conducted after the conclusion of the outbreak; it is conceivable that public perceptions and attitudes toward the use of restrictive measures could be different during the course of an outbreak. Finally, a relatively small proportion of our survey respondents were directly affected by quarantine during SARS, which precluded any analysis of differences between those who were directly affected and those who were not.

## Conclusions

The use of restrictive measures such as quarantine draws into sharp relief the push and pull of opposing forces that characterize the dynamic interplay between the personal autonomy of the citizen on the one hand and the collective rights of the community on the other. As Bensimon and Upshur [3] have argued, justification for quarantine cannot be founded upon scientific evidence alone; rather, the decision to implement quarantine should be equally informed by the values, preferences, and practices of the affected communities. The present findings indicate strong public support for the use of quarantine in the context of an infectious disease outbreak and for serious sanctions against those who fail to comply. Our data further suggest, however, that public support for quarantine is contingent on the implementation both of legal safeguards to protect against inappropriate use and of psychosocial supports to provide for individuals who are adversely affected. This tension between individual rights and the greater public good is precisely the challenge that infectious disease presents to public health ethics. In order to engender strong public support for the use of quarantine and other restrictive measures, government officials and public health policy-makers would do well to implement a comprehensive system of supports and safeguards, to educate and inform frontline public health workers, and to engage the public at large in an open dialogue on the ethical use of restrictive measures during infectious disease outbreaks.

## Competing interests

The authors declare that they have no competing interests.

## Authors' contributions

CST performed the statistical analysis of the survey data, drafted the first version of the manuscript, and contributed to subsequent revisions. ER initiated the study, participated in the design of the survey instrument, and contributed to the revising of the manuscript. REGU participated in the statistical analysis, contributed to the revising of the manuscript, and will act as guarantor. All authors have read and approved the final version of the manuscript.

## Pre-publication history

The pre-publication history for this paper can be accessed here:

http://www.biomedcentral.com/1471-2458/9/470/prepub

## References

[B1] Mandavilli A SARS epidemic unmasks age-old quarantine conundrum Nat Med 2003 9 487 10.1038/nm0503-487 12724741 PMC7095793

[B2] Bayer R Colgrove J Public health vs. civil liberties Science 2002 297 1811 10.1126/science.1074274 12228702

[B3] Bensimon CM Upshur RE Evidence and effectiveness in decision-making for quarantine Am J Public Health 2007 97 Suppl 1 S44 48 10.2105/AJPH.2005.077305 17413076 PMC1854977

[B4] Cetron M Landwirth J Public health and ethical considerations in planning for quarantine Yale J Biol Med 2005 78 329 334 17132339 PMC2259156

[B5] Gostin LO Sapsin JW Teret SP Burris S Mair JS Hodge JG Jr Vernick JS Balancing public health and civil liberties Science 2002 298 2129 author reply 2129 10.1126/science.298.5601.2129c 12481781

[B6] Schabas R Severe acute respiratory syndrome: Did quarantine help? Can J Infect Dis Med Microbiol 2004 15 204 18159492 10.1155/2004/521892 PMC2094974

[B7] Schabas R Is the Quarantine Act relevant? CMAJ 2007 176 1840 1842 17576981 10.1503/cmaj.070130 PMC1891118

[B8] Markel H Lipman H Navarro J Sloan A Michalsen J Stern A Cetron M Nonpharmaceutical interventions implemented by U.S. cities during the 1918-1919 influenza pandemic JAMA 2007 298 644 654 10.1001/jama.298.6.644 17684187

[B9] Hatchett RJ Mecher CE Lipsitch M Public health interventions and epidemic intensity during the 1918 influenza pandemic Proc Natl Acad Sci USA 2007 104 7582 7587 10.1073/pnas.0610941104 17416679 PMC1849867

[B10] Pang X Zhu Z Xu F Guo J Gong X Liu D Liu Z Chin DP Feikin DR Evaluation of control measures implemented in the severe acute respiratory syndrome outbreak in Beijing, 2003 JAMA 2003 290 3215 3221 10.1001/jama.290.24.3215 14693874

[B11] Svoboda T Henry B Shulman L Kennedy E Rea E Ng W Wallington T Yaffe B Gournis E Vicencio E Basrur S Glazier RH Public health measures to control the spread of the severe acute respiratory syndrome during the outbreak in Toronto N Engl J Med 2004 350 2352 2361 10.1056/NEJMoa032111 15175437

[B12] Day T Park A Madras N Gumel A Wu J When is quarantine a useful control strategy for emerging infectious diseases? Am J Epidemiol 2006 163 479 485 10.1093/aje/kwj056 16421244 PMC7109638

[B13] Hsieh YH King CC Chen CW Ho MS Hsu SB Wu YC Impact of quarantine on the 2003 SARS outbreak: a retrospective modeling study J Theor Biol 2007 244 729 736 10.1016/j.jtbi.2006.09.015 17055533 PMC7094157

[B14] Cava MA Fay KE Beanlands HJ McCay EA Wignall R Risk perception and compliance with quarantine during the SARS outbreak J Nurs Scholarsh 2005 37 343 347 10.1111/j.1547-5069.2005.00059.x 16396407

[B15] Robertson E Hershenfield K Grace SL Stewart DE The psychosocial effects of being quarantined following exposure to SARS: a qualitative study of Toronto health care workers Can J Psychiatry 2004 49 403 407 15283537 10.1177/070674370404900612

[B16] Hawryluck L Gold WL Robinson S Pogorski S Galea S Styra R SARS control and psychological effects of quarantine, Toronto, Canada Emerg Infect Dis 2004 10 1206 1212 15324539 10.3201/eid1007.030703 PMC3323345

[B17] Tansey CM Louie M Loeb M Gold WL Muller MP de Jager J Cameron JI Tomlinson G Mazzulli T Walmsley SL Rachlis AR Mederski BD Silverman M Shainhouse Z Ephtimios IE Avendano M Downey J Styra R Yamamura D Gerson M Stanbrook MB Marras TK Phillips EJ Zamel N Richardson SE Slutsky AS Herridge MS One-year outcomes and health care utilization in survivors of severe acute respiratory syndrome Arch Intern Med 2007 167 1312 1320 10.1001/archinte.167.12.1312 17592106

[B18] Statistics Canada Annual demographic estimates: census metropolitan areas, economic regions, and census divsions, age, and sex (2002 to 2007) http://www.statcan.ca/english/freepub/91-214-XIE/2007000/tablelist1.htm

[B19] Gostin L Public health strategies for pandemic influenza: ethics and the law JAMA 2006 295 1700 1704 10.1001/jama.295.14.1700 16609092

[B20] Blendon RJ Benson JM DesRoches CM Raleigh E Taylor-Clark K The public's response to severe acute respiratory syndrome in Toronto and the United States Clin Infect Dis 2004 38 925 931 10.1086/382355 15034821

[B21] DiGiovanni C Conley J Chiu D Zaborski J Factors influencing compliance with quarantine in Toronto during the 2003 SARS outbreak Biosecurity and Bioterrorism: Biodefense Strategy, Practice, and Science 2004 2 265 272 10.1089/bsp.2004.2.265 15650436

[B22] Blendon R Desroches C Cetron M Benson J Meinhardt T Pollard W Attitudes toward the use of quarantine in a public health emergency in four countries Health Affairs 2006 25 w15 w25 10.1377/hlthaff.25.w15 16434437

[B23] Gostin LO Bayer R Fairchild AL Ethical and legal challenges posed by severe acute respiratory syndrome: implications for the control of severe infectious disease threats JAMA 2003 290 3229 3237 10.1001/jama.290.24.3229 14693876

[B24] Annas GJ Bioterrorism, public health, and human rights Health Aff 2002 21 94 97 10.1377/hlthaff.21.6.94 12442843

[B25] Upshur RE Principles for the justification of public health intervention Can J Public Health 2002 93 101 103 11968179 10.1007/BF03404547 PMC6979585

[B26] Ries NM Public health law and ethics: lessons from SARS and quarantine Health Law Rev 2004 13 3 6 15838998

[B27] Singer PA Benatar SR Bernstein M Daar AS Dickens BM MacRae SK Upshur RE Wright L Shaul RZ Ethics and SARS: lessons from Toronto BMJ 2003 327 1342 1344 10.1136/bmj.327.7427.1342 14656848 PMC286332

[B28] University of Toronto Joint Centre for Bioethics Stand on guard for thee: ethical considerations in preparedness planning for pandemic influenza http://www.utoronto.ca/jcb

[B29] Joint Centre for Bioethics Pandemic Ethics Working Group Upshur R Public engagement on social distancing in a pandemic: a Canadian perspective Am J Bioeth 2009 9 11 15 17 10.1080/15265160903197598 19882445

